# Applications of Wearable Sweat Biosensors in Sports Activities with Real-World Cases

**DOI:** 10.3390/bioengineering13080906

**Published:** 2026-08-11

**Authors:** Jiaxiang Yan, Jingyu Shi, Zhuoer Zhang, Mo Yang, Ming Zhang, Meizi Wang

**Affiliations:** 1 Department of Biomedical Engineering, The Hong Kong Polytechnic University, Kowloon, Hong Kong 999077, China; jiaxiang.yan@polyu.edu.hk (J.Y.); jing-yu.shi@polyu.edu.hk (J.S.); zhuoer.zhang@connect.polyu.hk (Z.Z.); mo.yang@polyu.edu.hk (M.Y.); ming.zhang@polyu.edu.hk (M.Z.); 2 The Hong Kong Polytechnic University Shenzhen Research Institute, Shenzhen 518000, China; 3 Research Institute for Sports Science and Technology, The Hong Kong Polytechnic University, Kowloon, Hong Kong 999077, China; 4 Joint Research Center of Biosensing and Precision Theranostics, The Hong Kong Polytechnic University, Kowloon, Hong Kong 999077, China; 5 Research Centre for Nanoscience and Nanotechnology, The Hong Kong Polytechnic University, Kowloon, Hong Kong 999077, China

**Keywords:** sports injury risk, dehydration, sweat loss, wearable sweat sensors, machine learning, fatigue monitoring

## Abstract

Sports teams are constantly seeking advanced technologies to gain a competitive edge by enhancing athletic performance, accelerating recovery, and minimizing injury risk. Sweat serves as a readily accessible biofluid which enables non-invasive measurement of key biochemical analytes and physiological parameters during sports activities, providing valuable insights into an athlete’s fatigue status, hydration levels, energy expenditure, and muscle function. With ongoing advancements in wearable sweat sensors, particularly those powered by artificial intelligence (AI), these devices facilitate dynamic adjustments to training intensity, optimized fatigue management, personalized recovery strategies, and overall performance enhancement, which are especially valuable for athletes and sports teams. In this paper, we present a structured narrative overview of sweat-based wearable biosensor technologies, highlighting target analytes of interest, advanced materials, innovative designs, AI integration for data interpretation, and applications of commercial products in sports scenarios. Furthermore, we highlight challenges like sweat composition variability, calibration issues, and limitations in sensor materials. We also explore opportunities for future development, such as enhancing sensor performance with hybrid materials, expanding multimodal biosensors, and refining AI systems for personalized performance optimization.

## 1. Introduction

In both athlete and recreational sports activities, particularly for prolonged-endurance and high-intensity events, such as marathon running, ultramarathons, long-distance cycling, tennis, soccer, American football, and basketball, and intense multi-hour training sessions, maintaining an optimal physiological state is essential to sustaining performance and minimizing injury risk [1,2]. During prolonged athletic exertion, the human body undergoes significant physiological changes, including fluid depletion (dehydration), substantial losses of essential electrolytes (e.g., sodium, potassium, and magnesium) through sweating, alongside alterations in lactate metabolism [3]. Lactate accumulation is influenced by both exercise intensity and duration, reflecting the dynamic balance between lactate production and clearance. During submaximal aerobic efforts, improving lactate clearance efficiency as a function of exercise intensity and time is an important training goal for athletes, commonly addressed through lactate threshold training [4]. These physiological alterations could further lead to neuromuscular fatigue, exercise-associated muscle cramps, and an elevated risk of non-contact injuries, especially in the later stages of a race or session [5,6,7]. Therefore, the real-time monitoring of athletes’ physiological status has emerged as a key priority in evidence-based training program development. Such monitoring enables the scientific optimization of training schedules, better fatigue management, and reduced injury risks.

Human body fluids contain abundant biological information and serve as valuable sources of biomarkers for sports monitoring. Although blood has traditionally been regarded as the gold standard for biomarker analysis [8,9,10], its invasive sampling process can cause discomfort and precludes continuous monitoring. In contrast, non-invasive biofluids such as sweat, tears, and saliva offer more convenient alternatives. Among them, sweat is particularly attractive from an invasiveness and sampling standpoint, because it is naturally secreted onto the skin surface, enabling continuous detection, easy access, and efficient sampling at convenient body locations [11]. However, from a physiological perspective, accurately linking sweat biomarker concentrations to systemic physiological states remains a significant challenge due to factors such as sweat rate dependency and local gland metabolism [12].

Despite these challenges, sweat contains a variety of physiologically relevant biomarkers, including lactate, glucose, cortisol, and electrolytes, which have been shown to be valuable for assessing muscle fatigue, electrolyte balance, aerobic/anaerobic capacity, and hydration status during sports activities [13,14]. For example, moderate dehydration has been reported to reduce total distance covered and sport-specific skill performance by 13–15% in soccer players [15]. Since sweat rate and sweat electrolyte concentration differ among athletes, monitoring these parameters can help assess individual fluid and electrolyte losses during exercise [16]. Such information can be used to guide individualized carbohydrate electrolyte fluid replacement, helping athletes maintain hydration balance, sustain sprint performance, and preserve shooting accuracy [16]. This is especially important during extended matches or training sessions in sports such as tennis, football, soccer, and basketball, particularly under hot summer conditions or in poorly ventilated indoor environments, where sweat loss can increase rapidly. Therefore, continuous sweat monitoring could provide valuable information for evaluating hydration status, guiding personalized fluid and electrolyte intake strategies, and supporting decisions regarding an athlete’s readiness to re-enter play. To translate these physiological insights into practical sports monitoring applications, reliable wearable platforms capable of continuously collecting and analyzing sweat in real time are required.

Sweat biosensors have been developed using techniques such as electrochemistry, colorimetry, fluorescence colorimetry, and surface-enhanced Raman spectroscopy (SERS). A landmark advance that catalyzed this field was the development of soft, skin-interfaced epidermal microfluidic (“epifluidic”) patches by Rogers and co-workers, which employ capillary-driven PDMS microchannels and reservoirs to harvest, route, and store microliter volumes of sweat while minimizing evaporation and contamination [17]. When coupled with colorimetric assays, these platforms enabled battery-free, instrument-free, and simultaneous visual quantification of multiple analytes, including chloride, pH, glucose, and lactate, via simple smartphone image capture [18]. This convergence of controlled microfluidic sampling and multiplexed colorimetric readout established a foundational design paradigm that has since driven both academic innovation and commercial translation of wearable sweat sensors. Advancements in artificial intelligence (AI) and materials science have enhanced these sensors, supporting efficient, continuous, and real-time fatigue monitoring during training [19,20]. This enables training plan optimization, performance improvement, and injury risk reduction during exercise or competition [21]. For instance, AI-integrated smartphones can process sweat data for personalized insights.

While a growing body of literature has reviewed the rapid development of wearable sweat sensors, most of these studies have primarily focused on materials science, fabrication techniques, and electrochemical performance. However, a significant translational gap remains between bioelectronic engineering and practical sports physiology. Therefore, this narrative review aims to provide a comprehensive overview of recent advancements in AI-powered sweat-based wearable biosensors for physiological monitoring. Specifically, we emphasize their application in sports by tracking key metrics such as fatigue status, hydration levels, and energy expenditure. By synthesizing current evidence and outlining future directions, we address the challenges and opportunities of integrating these technologies into real-world training environments, highlighting their potential to enhance athletic performance and recovery through the continuous assessment of biochemical load responses.

### Review Approach and Literature Identification

This article was designed as a focused narrative review. The relevant literature was identified through searches of PubMed, Web of Science, Scopus, and Google Scholar completed on 20 January 2026, supplemented by the backward citation searching of relevant articles. The search concepts combined terms related to sweat (“sweat” and “perspiration”), wearable sensing (“wearable biosensor”, “microfluidic patch”, and “electrochemical sensor”), target analytes (“lactate”, “glucose”, “cortisol”, “sodium”, “potassium”, and “chloride”), artificial intelligence (“machine learning” and “artificial intelligence”), and sports applications (“exercise”, “athlete”, “training”, and “sport”). Peer-reviewed studies were prioritized when they addressed biomarker physiology, sensor development or validation, AI-assisted analysis, or applications during exercise. Official manufacturer, sports organization, and professional-team webpages were additionally consulted to identify product specifications and reported real-world deployment. Evidence was synthesized thematically into biomarker physiology, wearable sensing technologies, AI integration, and real-world sports applications. Because this was a narrative review, study selection was purposive, and no formal meta-analysis or risk-of-bias scoring was undertaken. The credibility of the sources, contextual clarity, logical organization of the evidence, and correspondence between the evidence and conclusions were examined with reference to the JBI Checklist for Textual Evidence: Narrative [22].

## 2. Key Biomarkers Found in Sweat During Sports Activities

Before examining individual biomarkers, it is essential to understand how analytes enter sweat, as this fundamentally governs their physiological interpretability. Eccrine sweat is generated through a two-stage partitioning process: molecules first diffuse from blood capillaries into interstitial fluid and are subsequently transported into the sweat gland lumen before secretion onto the skin surface [23]. As established in the biophysical framework proposed by Heikenfeld and colleagues, the efficiency of this transfer is strongly modulated by the analyte’s molecular weight, charge, lipophilicity, and the local sweat secretion rate [24,25]. Consequently, sweat biomarker concentrations rarely mirror blood levels in a simple one-to-one manner. While sweat offers ideal non-invasive and continuous access, this partitioning process often results in dilution, chemical modification, and temporal lags relative to systemic circulation. This represents a central challenge when interpreting sweat-based measurements as proxies for physiological status.

Despite these challenges, sweat contains several critical biochemical markers, including lactate, glucose, cortisol, and electrolytes, which have proven valuable for assessing muscle fatigue and hydration status during sports activities. These biomarkers provide direct insights into an athlete’s energy metabolism, anaerobic threshold, stress response, and electrolyte balance, enabling early detection of fatigue, overtraining risks, and suboptimal recovery, thereby supporting optimized training intensity, performance enhancement, and individualized recovery strategies. In this section, we highlight the key biomarkers found in sweat that are the most relevant to human performance during sports activities: lactate, glucose, cortisol, and electrolytes (Table 1).

### 2.1. Monitoring Sweat Lactate Levels

Lactate is produced when pyruvate is reduced by lactate dehydrogenase (LDH) in the absence of sufficient oxygen, regenerating NAD^+^ from NADH to sustain glycolysis [26]. It plays three major physiological roles during exercise: serving as a gluconeogenic substrate, acting as a cell-signaling molecule, and providing an optimal fuel source for working muscles. Exercise intensity drives lactate accumulation in two phases: Lactate Threshold 1 (LT1), where Type IIa fibers begin contributing and lactate rises gradually as production exceeds clearance, and Lactate Threshold 2 (LT2), where Type IIx fibers dominate, causing a sharp increase in lactate production and rapid elevation in blood levels via monocarboxylate transporters [27]. Monitoring these dynamic changes offers crucial insights into an athlete’s muscular metabolic status, aerobic/anaerobic capacity, and exercise-induced muscle fatigue. Consequently, training intensities can be strategically regulated to minimize wasteful energy consumption, ensuring that endurance and peak performance are sustained over extended durations [28,29]. However, despite its immense diagnostic value, traditional lactate monitoring relies on invasive blood sampling, which is painful, discontinuous, and often restricted to delayed post-exercise analysis. To overcome these inherent limitations, continuous lactate analysis in sweat has emerged as a highly promising, non-invasive alternative.

It has been reported that sweat lactate concentrations are often substantially higher than blood lactate concentrations, typically ranging from 5 to 40 mmol/L in sweat versus 0.5 to 25 mmol/L in blood during intense exercise, primarily because sweat lactate is largely produced locally by eccrine gland metabolism, where blood glucose is converted into lactate via glycolysis within the gland itself, rather than arising from the direct filtration or clearance of blood lactate [30]. Accordingly, the relationship between sweat lactate and blood lactate remains controversial, although many studies have reported a strong correlation between the two during exercise [31,32,33], others have found no significant correlations [34,35]. These inconsistent findings may result from differences in measurement methods, sampling sites, sample size, and other experimental conditions, indicating that correlation and regression analyses should be interpreted in a context-specific manner. Moreover, because lactate exists predominantly as a negatively charged ion at physiological pH, it cannot freely cross cell membranes; together with the time required for sweat production and transport to the skin surface, this introduces a physiological delay between muscle lactate production and its appearance in sweat.

Despite these physiological complexities, continuous evaluation of sweat lactate remains highly relevant for assessing an athlete’s metabolic state. Rather than relying on absolute discrete concentrations (such as the traditional > 4.0 mmol/L blood fatigue threshold) [36], sports physiology has shifted towards tracking continuous physiological trends. Recent evidence demonstrates that the dynamic inflection points in sweat lactate can closely mirror systemic blood lactate thresholds (bLTs) during incremental exercise [37]. As exercise intensity increases and muscles transition through LT1 and LT2, the corresponding sympathetic nervous system activation and metabolic shifts systemically alter sweat gland activity and lactate secretion rates. Therefore, continuously monitoring the relative dynamic changes and sweat lactate threshold inflection points can serve as a practical, non-invasive physiological reference for identifying metabolic exertion and fatigue onset during sports activities. This lays the fundamental biological groundwork for the application of advanced continuous-monitoring technologies.

### 2.2. Monitoring Sweat Glucose Levels

Glucose plays a crucial role during exercise, as it is a primary energy source for skeletal muscles. During physical activity, glucose uptake into the muscles is facilitated by the GLUT4 transporter, which translocates to the muscle cell membrane in response to muscle contractions and exercise. This process is essential to providing energy to the muscles, particularly during prolonged or intense exercise, when muscle glycogen stores become depleted [38]. The regulation of muscle glucose uptake during exercise involves several processes, including glucose delivery, transport, and metabolism [39]. Blood flow increases significantly during exercise, contributing to the enhanced glucose delivery to muscles. This is particularly important as muscle contractions trigger GLUT4 translocation to the sarcolemma and T-tubules, where it can facilitate glucose uptake into the muscle cells [40].

Furthermore, glucose uptake can be influenced by exercise intensity, with higher intensity exercises leading to greater glucose demand and uptake by the muscles. In some cases, glucose metabolism may be rate-limiting during exercise, particularly when the muscle’s glycogen stores are depleted and the muscle relies more on blood glucose as a fuel source. When blood glucose levels decrease below a certain threshold, muscles are unable to obtain sufficient energy, leading to muscle fatigue, resulting in decreased strength and decline in performance [41,42]. Studies have demonstrated a strong relationship between sweat and blood glucose [43,44,45]. Changes in blood glucose levels impact the glucose concentration in sweat due to the vascular nature of eccrine glands and the osmotic mechanism of sweat secretion.Sweat glucose concentrations typically range from 0.01 to 0.2 mM, significantly lower than blood glucose levels, which range from 4 to 10 m [46]. Therefore, a sensitive device is required to accurately monitor glucose concentrations in sweat. Various wearable devices, such as flexible patches and tattoos, have been developed to collect sweat for glucose monitoring. These devices typically integrate electrochemical sensors that detect glucose concentrations via enzymatic reactions, such as those involving glucose oxidase (GOx) [46]. In athletic contexts, sweat glucose monitoring enables personalized interventions, such as timely carbohydrate intake to prevent hypoglycemia-induced fatigue. For endurance athletes, it could detect transitions from aerobic to anaerobic metabolism, while in team sports, it informs recovery protocols to optimize performance and reduce injury risk from metabolic stress.

### 2.3. Monitoring Electrolytes for Hydration Status and Muscle Fatigue

In addition to lactate and glucose, electrolytes are crucial substances that play a key role in maintaining water balance by regulating fluid distribution between intracellular and extracellular compartments, supporting electrochemical potentials, and facilitating nutrients uptake and waste removal [47]. Muscle tissue contains approximately 76% water, with electrolytes acting as primary regulators of osmotic water flux and hydration status [48]. The loss of electrolytes through sweat, combined with fluid imbalances, can lead to hypohydration (dehydration), which impairs exercise performance, thermoregulation, and recovery, potentially increasing risks of heat-related illnesses, reduced endurance, and cognitive deficits [49,50]. Research further links electrolyte imbalances to neuromuscular factors influencing performance, such as alterations in skeletal muscle membrane potential, excitationcontraction coupling, and the onset of exercise-induced muscle cramps [51].

Key electrolytes in sweat include sodium (Na^+^), chloride (Cl^−^), and potassium (K^+^). Typical concentrations during exercise vary by individual factors, sweat rate, and environment: Na^+^ and Cl^−^ often range within 10–90 mmol/L, K^+^ within 2–10 mmol/L (typically stable at ~2–8 mmol/L, similar to plasma), and Ca^2+^ within 0.3–2 mmol/L [4,16,47]. Na^+^ and Cl^−^ levels are sweat rate-dependent due to reabsorption in eccrine sweat ducts. At low sweat rates (<0.5 L/h), ducts efficiently reabsorb these ions via active transport (e.g., Na^+^-Cl^−^ cotransporters), yielding hypotonic sweat with lower concentrations. High rates (>1.5–2 L/h) overwhelm reabsorption, leading to higher excretion and isotonic/hypertonic sweat [52]. Heat acclimation improves ductal reabsorption, reducing Na^+^ losses, while “salty sweaters” maintain high excretion (>60 mmol/L), raising hyponatremia risk during prolonged activity [53,54]. K^+^ remains stable and independent of sweat rate, serving as a reliable marker for contamination or dilution, as muscle efflux balances Na^+^-K^+^ pump activity [55,56,57].

Monitoring electrolytes during exercise optimizes training, prevents injuries (e.g., cramps from dehydration), and tailors recovery, such as Na^+^/Cl^−^ replacement to restore balance and reduce soft-tissue risks [58]. K^+^ tracking aids fatigue assessment from extracellular shifts. Recent progress in wearable sweat electrolyte sensing is largely driven by potentiometric electrochemical approaches, with key innovations in microfluidic sampling and multiplexed integration for the real-time monitoring of hydration and electrolyte balance. A key challenge in sweat analysis is the dependence of ion concentration on sweat rate and the difficulty of sampling low sweat volumes under variable environmental and physiological conditions.

### 2.4. Monitoring Cortisol in Sweat

Cortisol is regulated by the hypothalamic–pituitary–adrenal (HPA) axis and is a key biomarker for physiological and psychological stress responses during exercise [59]. Acute elevations in cortisol can mobilize energy substrates, enhance cardiovascular function, and modulate immunity for immediate performance demands. However, sustained high levels, often caused by high training loads, poor recovery, or overtraining syndrome, lead to catabolic effects (e.g., muscle degradation, immunosuppression, and impaired adaptation), contributing to fatigue, performance decline, mood disturbances, and injury risk. Sweat enables non-invasive, continuous monitoring (typical range of ~0.02–0.4 µmol/L), correlates well with serum or saliva levels, and offers real-time insights without interrupting activity [60]. Elevated sweat cortisol reflects HPA activation during intense/prolonged exercise, peaking at around 40 min in constant-load protocols, helping distinguish acute stress from chronic dysregulation indicative of central fatigue or early overtraining.

Recent innovations have demonstrated promising capabilities in using graphene-based wearable sensors for cortisol monitoring. These sensors utilize the exceptional properties of graphene to provide sensitive and reliable measurements, allowing for the detection of cortisol fluctuations that correspond with both the diurnal cycle and acute stress responses. Such devices have shown strong correlations with serum cortisol levels, providing valuable insights into both physical and psychological stress during athletic performance. Moreover, wearable sensors based on molecularly imprinted polymer (MIP) technology have been developed for the stable and long-term monitoring of cortisol, with high specificity even in the presence of other sweat components such as glucose and lactic acid. These advancements allow for precise, non-invasive cortisol tracking, which can be particularly beneficial for managing training loads, improving recovery strategies, and preventing overtraining syndrome.

## 3. Wearable Sweat Biosensors for Detection of Different Fatigue Biomarkers

Consistently and objectively measuring fatigue in real-world settings remains challenging. Traditional chemical biomarker measurement methods, such as blood sampling, are often invasive and discontinuous. In contrast, sweat is an attractive alternative biofluid because it can be collected non-invasively during exercise and contains multiple analytes relevant to fatigue physiology, including lactate, glucose, cortisol, and electrolytes. Recent advances in wearable sweat biosensor technologies now enable the continuous, real-time monitoring of these fatigue-associated biomarkers directly from the skin, facilitating dynamic assessment of internal workload and recovery status. To improve usability in sports and field applications, modern wearable sweat biosensors integrate microfluidic sampling, flexible ion-selective electrodes, and wireless data transmission, enabling on-body, lab-free operation. Additionally, AI algorithms are increasingly being applied to interpret complex wearable sweat biosensor data and derive personalized fatigue metrics.

### 3.1. Wearable Sweat Biosensors for Lactate Detection

**Figure 1:**
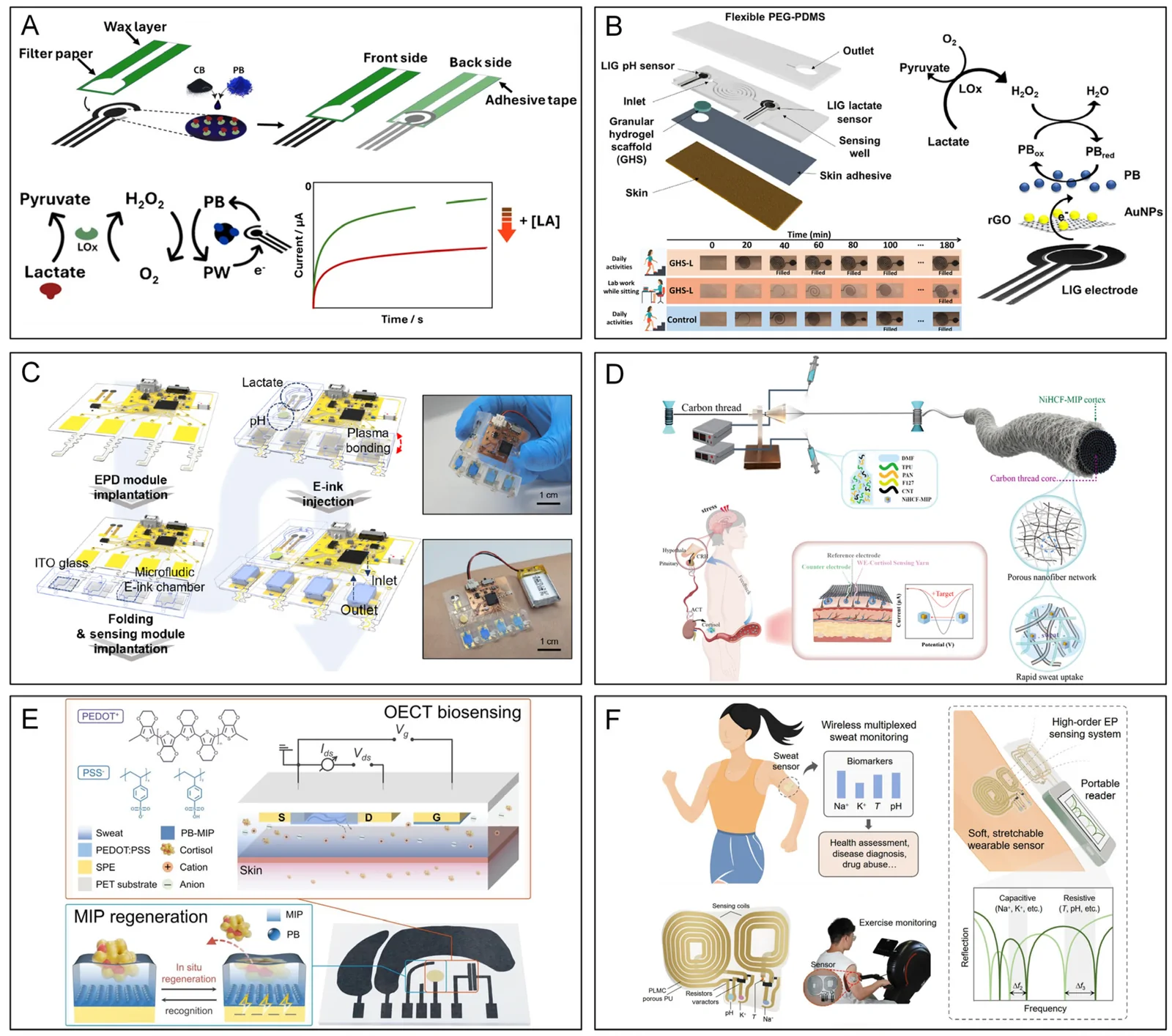
Representative schematic illustrations of advanced wearable sweat sensor architectures for non-invasive fatigue biomarker monitoring. (**A**–**C**) Lactate-focused electrochemical and hybrid platforms: 3D-printed flexible wearable biosensor for on-body sweat lactate monitoring; ref. [61] Copyright 2025, Springer Nature. Granular hydrogel-enhanced low-volume sampling system; ref. [62] Copyright 2025, Wiley-VCH GmbH. Electrophoretic digital colorimetry with on-skin E-ink display. Ref. [64] Copyright 2025, AAAS. (**D**) Dual-function core–shell nanocube probes enabling continuous production of cortisol-sensing yarns via conjugate electrospinning. Ref. [65] Copyright 2026, OAE Publishing Inc. (**E**) Computationally assisted MIP-OECT system for in situ receptor regeneration and continuous monitoring. Ref. [66] Copyright 2025, Elsevier. (**F**) Multiplexed stretchable wireless platform with exceptional-point resonators for simultaneous multi-biomarker sensing. Ref. [68] Copyright 2025, AAAS.

Recent progress in sweat lactate sensing is dominated by electrochemical approaches, with emerging hybrid systems incorporating optical readout for enhanced user accessibility. Meanwhile, to achieve the sensitivity and accuracy necessary for fatigue monitoring, advancements in sensing materials and fabrication have transitioned from planar electrodes to three-dimensional (3D) and nanostructured designs. For example, Iula et al. developed a 3D-printed flexible wearable biosensor for on-body sweat lactate monitoring (Figure 1A) [61]. The skin-conformal, lightweight patch integrates paper-based fluidics with screen-printed electrodes modified by a Prussian blue/carbon black biohybrid probe in a 3D-printed flexible arm band structure, enabling stable adhesion during vigorous movement, real-time lactate detection with good blood correlation, and practical continuous monitoring for athletic fatigue assessment. Lorestani et al. developed laser-induced graphene (LIG) electrodes by incorporating reduced graphene oxide–gold nanoparticle (rGO-AuNP) nanocomposites and a porous granular hydrogel scaffold (GHS) within a soft microfluidic patch (Figure 1B) [62]. This created a highly flexible system with strong skin adhesion that utilizes capillary and osmotic forces to achieve reliable sweat collection at rates as low as 119 nL min^−1^ cm^−2^. Even during rest or low-perspiration recovery, lactate levels can be monitored continuously without delamination or discomfort. Xuan et al. engineered an integrated, flexible wearable system featuring diffusion-limiting membranes to precisely control analyte flux and maintain a stable linear range [63]. The skin-interfaced patch uses advanced microfluidics, including a 3D-printed sweat-sampling cell with pressure control, to maintain constant fresh sweat flow over the electrodes, preventing the stagnation or mixing of old and new sweat, while demonstrating robust in vivo validation through strong correlations between sweat and blood lactate thresholds in elite athletes during dynamic exercises like cycling and kayaking. On the other hand, hybrid electrochemicaloptical systems have emerged for direct user feedback. Sung et al. developed an electrophoretic digital colorimetry platform that integrates enzymatic electrochemical sensing with a low-power E-ink display, converting lactate signals into on-skin color changes for real-time visual fatigue monitoring without external devices (Figure 1C) [64].

### 3.2. Wearable Sweat Biosensors for Cortisol Detection

Advances in wearable sweat-based cortisol sensing can be classified by transduction mechanism into colorimetric, electrochemical, and multiplexed electrochemical platforms, some of which integrate AI for enhanced analysis. Colorimetric approaches offer simple, equipment-free readouts suitable for field-based monitoring. Electrochemical approaches deliver higher sensitivity and enable continuous or real-time monitoring. For instance, Cheng et al. developed dual-function coreshell nanocube probes (NiHCF-MIP NCs) that enable continuous production of cortisol-sensing yarns via conjugate electrospinning (Figure 1D) [65]. This one-step coaxial electrospinning technique integrates redox-active nickel hexacyanoferrate cores with molecularly imprinted polymer (MIP) shells, yielding flexible, highly conductive, and sweat-absorbent sensing yarns. The resulting platform achieves high sensitivity (2.08 μA·dec^−1^) and a low limit of detection (0.4 nmol/L), supporting real-time, non-invasive sweat cortisol monitoring in wearable textile formats. Y. Liu et al. introduced a computationally assisted, flexible electrochemical platform integrating density functional theory-optimized molecularly imprinted polymers (MIPs with organic electrochemical transistors (OECTs) for in situ receptor regeneration (Figure 1E) [66]. This skin-adherent, low-power patch combines iontophoresis-induced sweat sampling and microfluidics for long-term circadian tracking, achieving ultra-low detection limits (0.36 nmol/L) with high selectivity against interferents (e.g., glucose and progesterone) and wireless signal transmission for potential closed-loop interventions. While sweat cortisol monitoring holds significant promise for fatigue assessment, especially when integrated with training load, recovery metrics, and additional biomarkers (e.g., lactate and cytokines), several challenges remain, including inter-individual variability, sweat rate dependence, and the need for personalized baselines. Emerging multimodal systems combining cortisol sensing with electromyography (EMG) of eye-blink analysis further offer enhanced predictive accuracy for overtraining risk [67].

### 3.3. Wearable Sweat Biosensors for Glucose Detection

In the field of wearable sweat glucose sensing, non-enzymatic electrochemical approaches have become predominant, aiming to overcome the inherent limitations of traditional enzymatic sensors. Non-enzymatic electrochemical approaches utilize nanocatalysts for direct glucose oxidation, providing enhanced stability against pH, temperature, and interferents in complex sweat matrices. For instance, Huang et al. integrated confined Pt nanoparticles (Pt-NPs) within 2D phthalocyanine-based metalorganic frameworks (Pc-MOFs), achieving high sensitivity (25.48 µA cm^−2^ mM^−1^), low limit of detection (LOD; 5.5 µM), and strong bloodsweat correlation (r = 0.968) over 12 h continuous monitoring with real-time pH/temperature calibration [69]. Yang et al. employed Au-NP-modified Ni-BDC nanoflowers in unidirectional microfluidic chambers with Tesla valves and integrated calibration sensors, enabling reliable in situ tracking via smartphone connectivity [70]. In addition to nanocatalyst-based sensors, emerging strategies explore direct electron transfer (DET) and biomimetic architectures to achieve next-level performance and energy autonomy. Qileng et al. developed Pt single-atom nanozymes that bypass H_2_O_2_ mediation to generate self-powered signals and achieve an ultra-low LOD (10 nM) during dynamic exercise [71]. Zhang et al. introduced ant-nest-structured multiwalled carbon nanotube sensors with pH-responsive colorimetric microchannels for rapid calibration and accurate pre- and post-diet glucose tracking [72]. In addition to electrochemical methods, optical approaches offer multiplexed, label-free detection for broader metabolite profiling. Wang et al. developed flexible, nanoplasmonic, paper-based sensors based on Ag nanoparticles on cellulose to generate SERS hotspots [73]. The soft, stretchable chip conforms naturally to skin, enabling rapid multiplexed detection (LOD 1 µM glucose) within 5 min through capillary-driven channels across pH 4–7.5.

### 3.4. Wearable Sweat Biosensors for Electrolyte Detection

For wearable sweat electrolyte monitoring, potentiometric electrochemical methods have become the primary driving force, complemented by key advances in microfluidic sampling and multiplexed integration for the real-time tracking of hydration and electrolyte balance. A key challenge in sweat analysis is the dependence of ion concentration on sweat rate and the difficulty of sampling low sweat volumes under variable environmental and physiological conditions. Microfluidic-enhanced potentiometric systems address these challenges by incorporating bio-inspired fluid-transport designs. For example, dendritic collectors with computational fluid dynamics (CFD)-optimized bifurcating channels enable rapid, efficient sampling even at low secretion rates, feeding into laser-engraved potentiometric ion-selective electrodes (ISEs) with near-Nernstian responses (67.3 mV/decade for Na^+^, 66.7 mV/decade for K^+^, and 38.2 mV/decade for Ca^2+^) [74]. Vertical fluidic-controlled bands transform sweat into uniform micro-droplets for dropwise detection, decoupling sweat rate from total electrolyte concentration via conductance analysis and eliminating long-channel artifacts [75].

### 3.5. Integrated Platforms for Multiplexed Biomarker Detection

A foundational route toward multiplexed sweat profiling was pioneered by John A. Rogers and colleagues through the development of soft, skin-interfaced epidermal microfluidic (“epifluidic”) platforms, which remain among the most widely translated technologies in the field [17,18]. These skin-conformal patches integrate networks of capillary-driven PDMS microchannels, valves, and colorimetric reservoirs that passively collect and compartmentalize sweat, enabling time-sequenced sampling without external power. When combined with chromogenic assays, they allow for simultaneous, instrument-free quantification of multiple analytes (e.g., chloride, pH, glucose, and lactate) and sweat rate via simple smartphone image analysis. This microfluidic–colorimetric paradigm effectively addressed long-standing challenges of sample evaporation, mixing, and contamination and has been successfully commercialized by Epicore Biosystems (see Section 4.2 for translational details).

**Figure 2:**
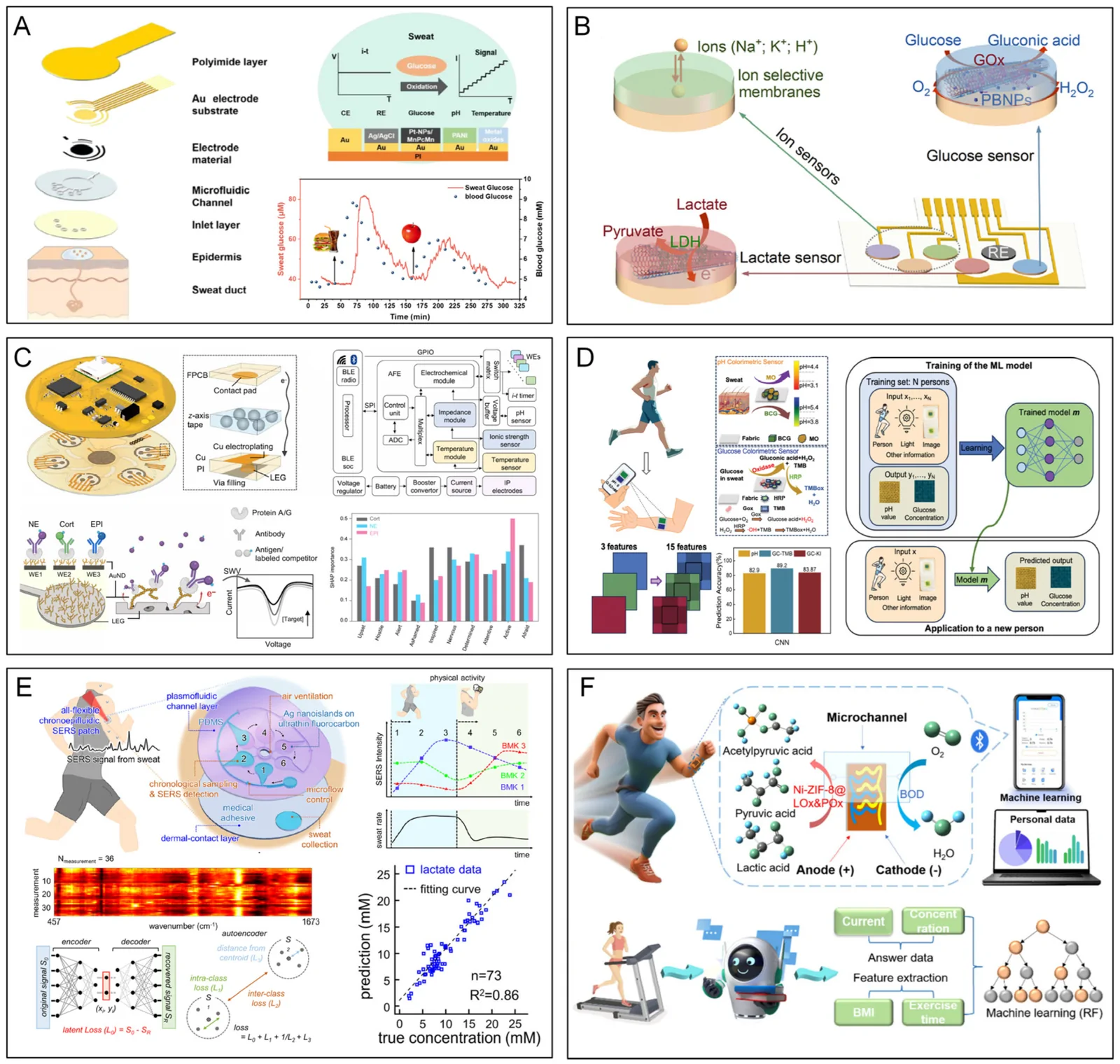
Conceptual framework of integrated AI-powered wearable sweat sensing systems for fatigue monitoring. The layered schematic illustrates the pipeline from skin-interfaced multiplexed hardware (top) through wireless multimodal data fusion to machine learning-driven predictive outputs (center and bottom). Hardware examples include (**A**) a skin-conformal Pc-MOF patch with confined Pt nanoparticles, integrated microfluidics, and real-time pH/temperature calibration for non-enzymatic glucose monitoring (Ref. [69] Copyright 2025, Wiley) and (**B**) an integrated wearable paper microfluidic array device for multiplexed in situ sweat analysis (Ref. [45] Copyright 2016, Springer Nature). The AI/ML processing and fusion layer includes (**C**) Random Forest models for early-window hormone-based state anxiety and affect prediction [76] (Copyright 2025, AAAS); (**D**) ML-enabled reliable colorimetric detection of pH and glucose via image/pattern recognition and classification (Ref. [77] Copyright 2025, Wiley); (**E**) an all-flexible chronoepifluidic nanoplasmonic patch for label-free SERS-based metabolite profiling in sweat, enhanced by ML-driven spectral analysis and feature extraction for improved biomarker identification and fatigue pattern recognition (Ref. [78] Copyright 2025, Springer Nature); and (**F**) an ML-assisted self-powered perspiration-driven sensor achieving 96.0% accuracy in real-time sweat lactate forecasting (Ref. [80] Copyright 2025, American Chemical Society).

Building upon this foundational microfluidic framework, subsequent efforts have evolved toward unified, electronically enhanced systems that enable the simultaneous monitoring of multiple biomarkers. With ongoing advances, wearable sweat sensors are shifting from single-analyte detection toward comprehensive physiological profiling, capturing real-time biochemical signatures of fatigue. By combining advanced microfluidics, array-based transducers, ion-selective membranes, and wireless connectivity, these platforms now provide rich, time-resolved, multi-analyte monitoring during exercise and recovery. Multiplexed potentiometric platforms extend capabilities to simultaneous multi-analyte tracking. For example, Ye et al. developed battery-free, skin-conformal patches based on third-order exceptional points (EPs) embedded in non-Hermitian resonators, which achieved over 10-fold sensitivity enhancements within an ultrathin format. Fabricated with strain-resilient liquid metal composites, the system supports simultaneous detection of Na^+^, K^+^, glucose, ammonium, and temperature, ensuring robustness under movement and strain. (Figure 1F) [68]. Huang et al. developed a flexible, skin-conformal wearable patch based on confined Pt nanoparticles (Pt-NPs) within 2D phthalocyanine-based metalorganic frameworks (Pc-MOFs) (Figure 2A) [69]. The thin-film device integrates microfluidic sampling channels with real-time pH and temperature calibration sensors, enabling 12 h continuous non-enzymatic glucose monitoring with high sensitivity (25.48 µA cm^−2^ mM^−1^), low LOD (5.5 µM), and strong bloodsweat correlation (r = 0.968). Another study by Yu et al. demonstrated the multiplexed in situ analysis of multiple sweat analytes (including Na^+^, K^+^, glucose, and lactate) using an integrated, wearable paper microfluidic array device for multicomponent electrochemical sweat sensing (Figure 2B) [45]. This low-cost, mass-producible platform leverages paper-based microfluidics with an array of electrochemical sensors, enabling efficient tandem sweat collection, rapid sampling, and accurate multi-analyte readings under varying exercise intensities and environmental conditions. Multiplexed electrochemical platforms further push boundaries by integrating high-sensitivity transduction with predictive analytics. The Stressomic system simultaneously measures cortisol, epinephrine, and norepinephrine using gold nanodendrite-decorated graphene electrodes and bursting valve-regulated microfluidics (picomolar sensitivity) [76]. This skin-conformal, battery-free device uses iontophoresis for on-demand sweat stimulation and supports wireless data transmission with picomolar sensitivity. Random Forest models trained on the first 20 min of time-series data achieved high predictive accuracy (86% for state anxiety, 62% for negative affect, and 54% for positive affect), enabling early differentiation of acute “fight-or-flight” responses from chronic allostatic load and prediction of psychological precursors to central fatigue.

### 3.6. AI Integration and Predictive Modeling

The advancement of continuous multi-biomarker sweat sensing has produced high-resolution time-series data on lactate, cortisol, glucose, and electrolytes, providing a strong foundation for integrating AI and machine learning (ML). Through multimodal data fusion, AI/ML enables accurate classification of fatigue states, real-time detection of physiological thresholds, and proactive alerts for overtraining, thereby transforming wearable sweat sensors from passive data acquisition tools into intelligent systems for decision making and personalized fatigue management.

Recent AI applications in sweat-based fatigue monitoring can be classified by algorithm type into ensemble methods, supervised learning models, and hybrid self-powered systems. Specifically, ensemble methods, such as Random Forest (RF), excel at noise robustness and featureimportance ranking for early psychological stress prediction. The Stressomic platform employs RF models trained on the first 20 min of multiplexed hormone dynamics (cortisol, epinephrine, and norepinephrine), achieving 86% accuracy in state anxiety classification and 62%/54% for negative/positive affect (Figure 2C) [76]. This early-window approach enables proactive alerts for psychological precursors of central fatigue and overtraining. Supervised learning models such as artificial neural networks (ANNs), support vector machines (SVMs), and gradient boosting handle high-dimensional multi-biomarker fusion for comprehensive fatigue profiling. Zhou et al. reported a colorimetric wearable sensor integrated with ML that enables reliable pH and glucose detection in sweat by processing smartphone-captured images to correct for lighting variations, color interpretation errors, and interferents, delivering high accuracy and robustness for real-time, user-friendly monitoring (Figure 2D) [77]. In parallel, emerging chronoepifluidic nanoplasmonic patches enable the label-free, time-sequential SERS-based profiling of multiple sweat metabolites (including glucose-relevant signals), where the high-dimensional spectral data are ideally suited for supervised ML models (e.g., ANN, SVM, or gradient boosting) to perform feature extraction, denoising, and fatigue-related temporal pattern recognition (Figure 2E) [78]. In the sweat-omics framework for elite soccer, these supervised models support multimodal fatigue mapping across neuromuscular, metabolic, inflammatory, and psychological domains, leveraging sweat biomarkers (e.g., lactate, sodium, cortisol, and IL-6) to identify patterns of accumulated fatigue and recovery dynamics. While the narrative review itself does not implement specific ANN, SVM, or gradient boosting models, it highlights the foundational role of advanced analytical platforms in processing complex sweat-omics data, providing a conceptual basis for ML-driven integration and the predictive modeling of multi-domain fatigue in high-performance athletes [19]. In parallel, ML-assisted platforms tracking glucose–cortisol interplay in passive sweat demonstrate high-fidelity temporal profiling across activity, nutrition, and circadian influences, enhancing metabolic-stress response insights [79]. Hybrid self-powered systems incorporate on-device ML for real-time compensation and forecasting. A perspiration-driven sensor with Ni-ZIF electrodes achieves 96.0% prediction accuracy for sweat lactate by correcting pH, temperature, and interferents, supporting automated exercise intensity zoning and early fatigue onset detection (Figure 2F) [80]. Wearable sweat biosensors designed to monitor different fatigue biomarkers are summarized in Table 2.

**Table 2:** Overview of representative wearable sweat biosensors for lactate, cortisol, glucose, and electrolytes in fatigue monitoring.

Biomarker	Detection Method	Performance Highlights	Multiplexing Capability	Fatigue/Relevance Aspect	Ref.
Lactate	Electrochemical (3D microstructured)	Sensitivity: 9.76 μA mM^−1^ cm^−2^, Range: 0.1–30 mM	Single (lactate)	Multi-day cumulative trend for residual muscle fatigue	[81]
Lactate	Electrochemical (LEG-based)	Simultaneous Na^+^, K^+^, lactate, sweat rate	Yes (Na^+^, K^+^, lactate, sweat rate)	Differentiates trained vs. untrained athletes	[82]
Lactate	Electrochemical (LIG + rGO-AuNP + GHS)	15-fold sensitivity increase, Sampling: 119 nL min^−1^ cm^−2^	Single (lactate)	Continuous monitoring in low-perspiration/rest phases	[62]
Lactate	Electrochemical (diffusion-limiting)	In vivo validation vs. blood LT in elite athletes	Single (lactate)	Precise correlation with physiological thresholds	[33]
Lactate	Electrochemical + ML (self-powered)	96.0% ML prediction accuracy	Single (lactate)	Early fatigue forecasting	[80]
Lactate	Hybrid electrochemical-optical (EPD)	Real-time visual feedback without external devices	Single (lactate)	Direct user visual fatigue monitoring	[64]
Cortisol	Colorimetric (LFA + AuNFs)	LOD ~75 pg/mL, matches saliva/serum	Single (cortisol)	Circadian rhythms, acute stress, jet lag	[83]
Cortisol	Electrochemical (MIP-OECT)	LOD 0.36 nmol/L, high selectivity vs. interferents	Single (cortisol)	Long-term circadian tracking, closed-loop potential	[66]
Cortisol	Multiplexed electrochemical + AI	Picomolar sensitivity, RF models: 86% state anxiety accuracy	Yes (cortisol + epinephrine + norepinephrine)	Early psychological fatigue/overtraining prediction	[76]
Glucose	Non-enzymatic electrochemical (Pc-MOFs)	Sensitivity 25.48 µA cm^−2^ mM^−1^, LOD 5.5 µM, r = 0.968 vs. blood	Single (glucose)	12-h continuous monitoring	[69]
Glucose	Non-enzymatic electrochemical (Ni-BDC)	Reliable in situ tracking, prevents backflow	Single (glucose)	Real-time during activity/daily routines	[70]
Glucose	Non-enzymatic (Pt single-atom nanozymes)	LOD 10 nM	Single (glucose)	Dynamic exercise monitoring	[71]
Glucose	Biomimetic electrochemical (MWCNT ant-nest)	Accurate pre-/post-diet tracking	Single (glucose)	Good blood-sweat correlation	[72]
Glucose	Optical (nanoplasmonic SERS)	LOD 1 µM glucose, multiplexed in 5 min, pH 4–7.5	Yes (glucose + uric acid + pH)	Multiplexed metabolite profiling	[73]
Electrolytes	Potentiometric (dendritic collectors + ISEs)	Near-Nernstian responses (67.3–38.2 mV/decade)	Yes (Na^+^, K^+^, Ca^2+^)	Reliable multi-ion tracking	[74]
Electrolytes	Conductance-based (vertical fluidic)	Decouples rate from concentration	Yes (total electrolytes)	Accurate hydration monitoring	[75]
Electrolytes	Multiplexed potentiometric (EP-based)	>10-fold sensitivity, simultaneous Na^+^/K^+^/glucose/ammonium/temperature	Yes (Na^+^, K^+^, glucose, ammonium, temperature)	Prolonged wear with wireless monitoring	[68]

## 4. Application of Wearable Sweat Biosensors in Sports Activities in Real Cases

Athletes, coaches, and sports science researchers are continuously looking for advanced technologies to improve athletic performance, prevent injuries, and optimize training schedules to gain a competitive edge on the field. Over the last decade, physiological state monitoring methods for athletes, such as invasive blood sampling and periodic heart rate assessments in controlled laboratory settings, have been commonly used. While these traditional methods were once considered the state of the art, they faced some critical limitations, including invasiveness and discomfort, highly labor-intensive data collection, and inability to capture key real-time physiological parameters or biomarkers which are essential to monitoring athletes’ health and performance. Recent achievements in wearable biosensor technology, from compact and portable devices to integrated and AI-powered system, have provided new paradigms for data collection and interpretation and are now being implemented in sports worldwide. In this section, we highlight the application of wearable sweat sensors by discussing commercial devices to measure crucial analytes monitoring human physiology state during sports activities. Table 3 provides an overview of various sweatmonitoring products.

### 4.1. Epicore Gx Sweat Patch

**Figure 3:**
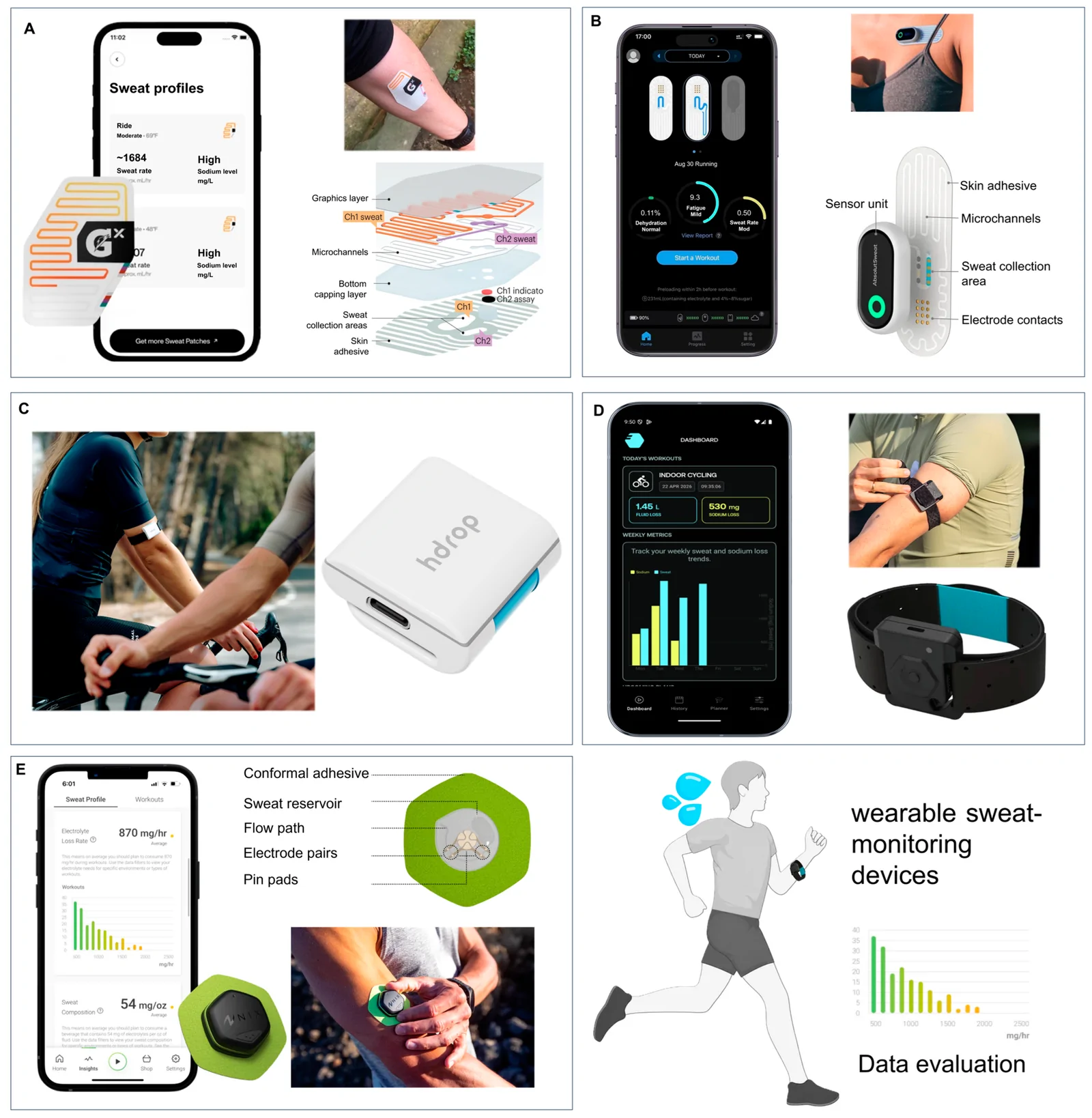
**Overview of commercial wearable sweat sensors for athletic monitoring.** Overview of commercial wearable sweat sensors for athletic monitoring. (**A**) Epicore Gx Sweat Patch (Epicore Biosystems, Cambridge, MA, USA, in partnership with Gatorade/PepsiCo). Image courtesy of Liipoo; reproduced with permission [86]. (**B**) Liipoo AbsolutSweat S1 (Liipoo, Shenzhen, China). Image courtesy of Liipoo; reproduced with permission [90]. (**C**) hDrop Gen 2 (hDrop Technologies, Little Rock, AR, USA). Image courtesy of Liipoo; reproduced with permission [91]. (**D**) FLOWBIO S1 Sweat Sensor (FLOWBIO, London, UK). Image courtesy of Liipoo; reproduced with permission [92]. (**E**) Nix Hydration Biosensors (Nix Biosensors, MA, USA); reproduced with permission [94].

Developed through a collaboration between Epicore Biosystems and The Gatorade Company, the Gx Sweat Patch represents one of the earliest clinically validated consumer wearable sweat sensors (Figure 3A) [86]. It primarily measures local sweat rate (LSR) and sweat chloride concentration ([Cl^−^]) via microfluidic colorimetric analysis, with app-based algorithms predicting whole-body sweat rate, fluid loss, and sodium loss for personalized hydration guidance.

Three large clinical trials have been published, demonstrating the use of the Gx Sweat Patch to monitor sweating rate, sweat [Cl^−^], and total sweat loss, with validation against standard absorbent patch methods in various sports scenarios. For example, Baker et al. published a research paper in 2020, where they tested 312 competitive athletes across a range of sports, including stationary cycling, soccer, tennis, lacrosse, and track and field, and demonstrated stronger correlations between the Gx patch and the standard method in the metrics of local sweat rate and sweat [Cl^−^] [87]. The second study aimed to validate the Gx Sweat Patch under a wider range of conditions, targeting recreational exercisers in cooler environments (down to 8 °C) and sports activities like fitness and running [88]. The device exhibited good day-to-day reproducibility, with coefficients of variation (CV) of 9–13% for local sweat rate (LSR) and 11–14% for sweat [Cl^−^]. The third study validated the Gx Sweat Patch in uncontrolled G-League basketball training, confirming correlations with absorbent patches and applicability to elite players (tattooed/untattooed) during moderate-intensity training sessions. No significant differences were observed between predicted and measured whole-body sweat rate, and 71% of predictions fell within ±0.5% body mass [89]. All of these studies showed significant correlations between the Gx Sweat Patch and absorbent patch methods across different sports and environmental conditions. They highlighted the robustness of the app-based algorithms to predict whole-body sweat rate and sweat [Na^+^]. This supports the use of the Gx Sweat Patch as an effective real-time tool for monitoring athletes’ physiological status and informing dynamic fatigue management. However, an important limitation is patch retention during high-contact sports (e.g., basketball scrimmages or football), where dropout rates reached ~14%, compared with only 2–5% in low-contact activities [89].

### 4.2. Liipoo AbsolutSweat S1

The AbsolutSweat S1 (developed by Liipoo, a Shenzhen-based sports tech company founded in 2016 and specializing in wearable fitness devices) uses a clip-on sensor that attaches to a disposable adhesive sweat patch (worn on the chest or other areas) to collect and analyze sweat via electrochemical biosensing, measuring biomarkers such as sweat rate, fluid loss (L/h), sodium (Na^+^), potassium (K^+^), glucose, and potentially other metabolites (e.g., lactate in advanced modes) and then transmitting data via Bluetooth to the AbsolutSweat app for personalized hydration guidance, electrolyte replenishment alerts, fatigue estimation, and dehydration tolerance tracking (Figure 3B) [90]. Luo et al. conducted a feasibility study using the AbsoluteSweat brush-chip smart sweat analyzer, a wearable device applied to the chest to monitor sweat Na^+^ and K^+^ concentrations in real time during an incremental cycling test in 55 male participants [84]. The sensor provided minute-by-minute data, allowing for identification of the sweat sodium threshold (sST) and the sweat potassium threshold (sPT) as proxies for AT. The results showed strong correlations between these sweat thresholds and the blood lactate threshold (bLT) in medium- and high-fitness groups. This application highlights how wearable sweat sensors can link sweat ion dynamics to metabolic transitions, such as K^+^ efflux during intense exercise, offering a practical alternative for endurance sports monitoring without blood draws.

### 4.3. hDrop Gen 2 and FLOWBIO S1 Sweat Sensor

The hDrop Gen 2 (developed by hDrop Technologies) is a reusable, non-invasive wearable sweat sensor designed as a compact and skin-contact device for the real-time monitoring of hydration-related biomarkers during exercise (Figure 3C) [91]. The hDrop Gen 2 employs patented carbon-printed electrode technology in direct skin contact to detect sweat analytes electrochemically. This enables continuous estimation of sweat rate (L/h), total sweat loss, sodium (Na^+^) and potassium (K^+^) losses, and near-skin temperature, with data streamed via Bluetooth to a companion smartphone app for personalized hydration recommendations (e.g., fluid volume and electrolyte replenishment timing/amount). This is in partnership with RCD Espanyol LaLiga soccer club for player training and hydration programs.

The FLOWBIO S1 Sweat Sensor (developed by FLOWBIO, a London-based company) is a reusable, non-invasive wearable hydration-monitoring device designed for real-time analysis of sweat during exercise (Figure 3D) [92]. It clips onto standard heart rate chest straps, arm bands, or compatible gear (e.g., back, upper-arm, triceps, or forearm positions) and uses microfluidic channels combined with electrochemical conductivity sensors and a high-precision skin-temperature sensor to capture and analyze sweat. The device samples every 8 s, measuring local sweat rate, fluid loss, sodium concentration, and total sodium loss, and then extrapolates to whole-body estimates via proprietary algorithms. Bandiera et al. evaluated the validity of S1 Sensor (FLOWBIO) for measuring regional sweat [Na^+^] and estimating whole-body sweat loss against gold-standard methods in cycling. The results indicated that S1 offers more practical collection of sweat [Na+] during indoor cycling ergometer exercise [85].

### 4.4. Nix Hydration Biosensors

Launched on the market in 2022, the Nix Hydration Biosensor consists of a reusable electronic pod (rechargeable and lightweight at under 0.5 ounces) that clips onto a single-use adhesive patch worn on the bicep (Figure 3E). The patch uses proprietary electrochemical sensors to detect biomarkers in sweat, including fluid loss, electrolyte concentrations (primarily [Na^+^], with derived [K^+^] and [Cl^−^] insights), sweat rate (L/h), total sweat loss, and skin temperature [93]. Data are transmitted in real time via Bluetooth to the Nix Solo mobile app or Nix Pro platform (designed for group team use). ML algorithms predict local arm measurements to whole-body estimates, providing dynamic notifications such as fluid intake volume, electrolyte replenishment needs, and alerts to maintain optimal hydration [94]. The Nix Hydration Biosensor has transitioned to elite sports with professional partnerships. In 2025, Chicago Fire FC Major League Soccer (MLS) became the first MLS team to use Nix biosensor for real-time sweat analytics during training and matches [95]. Specifically, it has been used to personalize hydration strategies and optimize training load management.

Additional high-profile partnerships include US Speedskating, LSU Football, Legacy Motor Club, and Union Cycliste Internationale (UCI) approval for professional cycling use [96]. These collaborations demonstrate the device’s reliability for dynamic athlete monitoring. By providing continuous, individualized hydration feedback, the Nix biosensor helps coaches identify early signs of electrolyte imbalance that may precede muscle cramps or the onset of peripheral fatigue.

### 4.5. ONASPORT Sweat Sensor

Another wearable sweat device such as The Onasport was developed by Onalabs Inno-Hub SL, which specializes in non-invasive biosensors for sports and healthcare (Figure 3) [97]. It uses a reusable central module integrated into a chest strap with disposable microfluidic cartridges (ONAS10, single-use sweat collection patches) worn on the chest to collect and analyze sweat via patented electrochemical sensing, measuring biomarkers such as sweat rate, total sweat loss, sodium concentration, inferred lactate, dehydration estimate, and heart rate. Data transmit via Bluetooth Low Energy to the Onasport app for real-time insights, including personalized hydration plans, electrolyte replenishment alerts, lactate threshold identification, fatigue monitoring, and performance optimization during training or competition.

Aguilar-Torán et al. presented the foundational validation of this wearable system in a study developing a novel sweat-based device for athletic biometrics, tested in vitro and in vivo during exercise [98]. The prototype, worn as a chest strap, used microfluidic channels to continuously monitor sweat rate (via capacitive sensing), ionic conductivity (for Na^+^-dominated electrolyte estimation), lactate (via amperometric biosensor with lactate oxidase), and heart rate. In vivo trials during physical activity demonstrated reliable real-time data capture, with the system estimating dehydration from perspiration volume and ionic strength, and inferring blood lactate equivalents from sweat lactate, heart rate, and sweat rate correlations. The results showed the device’s accuracy in tracking metabolic shifts, such as lactate accumulation during anaerobic efforts, offering a non-invasive alternative to blood draws for assessing thresholds (e.g., LT1/LT2) and preventing overexertion. This highlights the potential of sweat sensors for linking ion and metabolite dynamics to physiological transitions in endurance sports, enabling proactive coaching without interrupting training.

Even though commercial products of wearable sweat biosensors have been widely used by elite athletes and professional teams in sports like soccer, triathlon, and cycling for real-time physiology state monitoring, they still face significant challenges. Sensor sensitivity remains limited when detecting ultra-low concentrations of biomarkers (e.g., metabolites or ions in trace sweat volumes), often requiring stimulation or high sweat flow that is not always available during low-intensity or non-exercise periods. Power consumption poses a major barrier for continuous conduction, as miniaturized devices must balance frequent sampling, wireless transmission, and long battery life without bulky power sources, especially under dynamic motion or variable environmental conditions. Data accuracy is frequently compromised by motion artifacts, skin contamination, biofouling, evaporation effects in humid environments, and inter-individual physiological variability (e.g., sweat composition changes with acclimation, intensity, or hydration status), leading to biases when compared with lab gold standards like flame photometry or scale-based sweat loss measurements. These issues highlight the need for further advancements in materials, low-power electronics, robust calibration algorithms, and large-scale field validations to achieve reliable, personalized performance insights across diverse sports and conditions.

## 5. Conclusions

Over the past decade, wearable sweat biosensor technologies have developed from the proof-of-concept laboratory demonstrations into commercial devices, showing real-world value in sports, allowing coaches and athletes to tailor training loads and recovery protocols based on personalized biochemical profiles. Innovations in materials science, microfluidics, flexible electronics, and combined AI data analyses have addressed many limitations noted in previous research, such as low sensitivity, signal noise, and static data interpretation [21]. Currently, those systems now routinely integrate multi-analyte with AI models that contextualize sweat biomarkers relative to personalized baselines, enabling real-time and continuous monitoring of the physiological state of athletes during exercise.

Despite these notable advances, significant challenges that limit the effectiveness of wearable sweat sensors in sports monitoring still remain. First, sweat composition is highly variable and can be influenced by sweat rate, temperature conditions, exercise intensity, and inter-individual differences. As highlighted in Table 1, achieving accurate monitoring is further complicated by the extreme concentration disparities among targets and the inconsistent between blood and sweat correlations. Although advanced algorithms can partially compensate for these variables, the lack of standardized calibration across users and conditions still affects sensor accuracy and biomarker correlations with blood or systemic levels. Materials and sensor design also continue to impose constraints. Current wearable platforms rely on soft elastomers and hydrophilic microchannels to collect and transport sweat, yet long-duration adherence during intense activity can cause skin irritation, delamination, or fluid leakage. Hybrid materials that integrate nanomaterials such as carbon nanotubes, metal organic frameworks, and advanced polymers have potential to improve the sensitivity and selectivity for multiple biomarker detection.

Additionally, from an analytical perspective, while AI-driven sensors have greatly enhanced signal and data interpretation, current models are largely trained on controlled datasets and may not generalize across various sports activities, environmental conditions, or ethnic groups with different sweat profiles. This calls for more diverse and longitudinal datasets that reflect real-world use cases, coupled with transparent and explainable AI systems that support actionable insights rather than opaque score outputs. Also, another challenge is to integrate sweat sensor output into holistic athlete monitoring frameworks. The requirement of coaches and sports scientists is not just limited to individual biomarkers but multimodal insights that combine sweat data with heart rate, muscle activation, and biomechanical metrics. For example, the International Olympic Committee (IOC) consensus statement on recommendations and regulations for sports events in the heat emphasizes the value of real-time technology to monitor athletes’ physiological and biomechanical performance indicators during competition, enabling prompt medical decisions to prevent injury and illness [83]. Integration of multiple sensors into a unified suite for the simultaneous tracking of various metrics supports this goal by providing timely, data-driven interpretation for performance optimization and athlete safety, particularly in high-risk heat environments.

## Figures and Tables

**Table 1:** The key biomarkers measured in sweat for monitoring the physiological state in humans during sports activities.

Biomarker	Typical Sweat Concentration (During Exercise)	Justification/Role in Athlete Performance	Recognition Element	Blood/Sweat Correlation
Lactate	5–20 mM (often higher than in blood; 5–50 mM in some contexts)	Workout intensity, anaerobic metabolism, and fatigue indicator (lactate inflection point)	Lactate oxidase (LOx)	Inconsistent/direct correlation; often reflects local gland production; some threshold alignment
Glucose	10–200 µM (0.01–0.2 mM; much lower than blood)	Energy substrate availability, fatigue from hypoglycemia/hyperglycemia	Glucose oxidase (GOx)	Moderate correlations in studies; variable due to low levels, dilution, and local metabolism
Cortisol	Low (e.g., 8–140 ng/L or ~0.02–0.4 µmol/L equivalents; trace levels)	Stress response (HPA axis), mental acuity, and overtraining/fatigue indicator	Not specified in detail (e.g., emerging: ZnO, MoS_2_, or polymeric for impedance/electrochemical)	Correlates with saliva/serum; larger inter-subject variation suggests high stress sensitivity
Na^+^	10–90 mmol/L (sweat rate-dependent; 10–100 mM)	Hydration status and electrolyte balance; prevents hypohydration/hyponatremia/cramps	Na ionophore (ion-selective membrane)	Good for plasma loss models; rate-adjusted metrics essential; high in “salty sweaters”
Cl^−^	10–90 mmol/L (sweat rate-dependent; 10–100 mM)	Hydration/electrolyte balance, linked to Na^+^ for fluid retention and cramping prevention	Ag/AgCl electrodes (or Cl-selective)	Strong dehydration link; similar rate dependency as Na^+^; used in cystic fibrosis adaptations
K^+^	2–10 mmol/L (often ~2–8 mmol/L, stable)	Muscle activity and membrane potential; stable marker for contamination/dilution	K ionophore (ion-selective membrane)	Similar to plasma; less rate-dependent; reliable for sample quality assessment

**Table 3:** Recent commercial wearable sweat-monitoring devices in sports scenarios.

Product	Manufacturer/Company	Type/Design	Key Measurements	Reusability/Cost Model	Validation/Accuracy Notes	Primary Use/Partnerships	Website
Nix HydrationBiosensor	Nix Biosensors (Boston, MA, USA)	Reusable rechargeable pod + single-use adhesive patch (upper arm)	Fluid/electrolyte loss, sweat rate/composition (Na^+^ focus)	Reusable pod (~$130 starter with 4 patches); consumable refills	Validated for real-time fluid/electrolyte tracking; reviews praise accuracy/trends; integrations with Garmin/Apple Watch	Endurance/team sports; Chicago Fire FC (MLS soccer), US Speedskating, and UCI cycling approval	https://nixbiosensors.com/ (accessed on 20 February 2026)
Epicore Gx Sweat Patch	Epicore Biosystems (Cambridge, MA, USA)/Gatorade (PepsiCo)	Disposable microfluidic colorimetric patch (arm) + app scanning	Sweat rate, total fluid loss, chloride/sodium concentration	Single-use patches (~$25 for 2); no reusable hardware	Clinically validated in blinded multi-sport studies (e.g., 312 athletes across cycling/soccer/tennis; strong correlations with absorbent patches)	Athletic performance/hydration; Gatorade partnership; used in soccer, cycling, and basketball	https://www.epicorebiosystems.com/ (accessed on 20 February 2026)
Liipoo AbsolutSweat S1	Liipoo (Shenzhen, China)	Reusable clip-on sensor + disposable adhesive patch (chest/arms)	Sweat rate, fluid loss (L/h), Na^+^/K^+^, glucose, and fatigue indicators	Reusable sensor (~$250–$400 starter kit); consumable patches	Used in 2025 feasibility study [84] (Luo et al.) for Na^+^/K^+^ during cycling; strong correlations with blood lactate in medium-/high-fitness groups	Athletes/fitness; endurance focus; endorsements from triathletes (e.g., Matt Hanson)	https://liipoo.com/ (accessed on 20 February 2026)
hDrop Gen 2	hDrop Technologies (Little Rock, AR, USA)	Reusable arm-worn sensor (direct skin contact, no patch)	Sweat rate/loss, Na^+^/K^+^ depletion, skin temperature	Fully reusable (~$250); no disposables	Preliminary lab validation (~92% agreement for sweat loss/Na^+^ in controlled protocols); field reviews note trend accuracy (some underestimation)	Endurance/team sports; partnerships with RCD Espanyol (LaLiga soccer) and IRONMAN Pro Series (Matt Hanson)	https://hdroptech.com/ (accessed on 20 February 2026)
FLOWBIO S1 Sweat Sensor	FLOWBIO (London, UK)	Reusable clip-on sensor (attaches to HR strap/arm band)	Sweat loss (L/h), sodium loss (mg/L), and skin temperature	Fully reusable (~£329); no disposables	Independent 2026 validation [85]): accurate WBSL vs. scale (bias +0.017 kg); [Na^+^] equivalent to LAQUAtwin but underestimated vs. flame photometry (~10 mmol/L bias)	Endurance/team sports (supports soccer/football modalities); trials in Australian soccer clubs	https://flowbio.com/ (accessed on 20 February 2026)
ONASPORT	Onalabs Inno-Hub SL (Cerdanyola del Vallès, Spain)	Wearable sweat biomarker sensor integrated in a chest strap format with heart-rate strap	Lactate (sweat-inferred), heart rate, sweat rate, dehydration, and sodium and electrolyte loss	Device reusable, but uses disposable ONAS10 sweat cartridges for each session	Validated in sports science centers (CAR Sant Cugat, INEFC, Clínica Ergodinámica) and piloted with endurance athletes; uses electrochemical microfluidic sensing and trained models to estimate internal load parameters in real time	Athlete training optimization and endurance performance monitoring; partnerships include CAR Sant Cugat, INEFC; integrations with Garmin	https://onalabs.com/en/onasport/ (accessed on 20 February 2026)

## Data Availability

No new data were created or analyzed in this study. Data sharing is not applicable to this article.
